# Systematic Review of Integrative Health Care Research: Randomized Control Trials, Clinical Controlled Trials, and Meta-Analysis

**DOI:** 10.1155/2011/636134

**Published:** 2010-09-08

**Authors:** Raheleh Khorsan, Ian D. Coulter, Cindy Crawford, An-Fu Hsiao

**Affiliations:** ^1^Military Medical Research and Integrative Medicine, Samueli Institute, 2101 East Coast Hwy, Corona Del Mar, CA 92625, USA; ^2^Department of Planning, Policy and Design, School of Social Ecology, University of California Irvine, Irvine, CA 92697-7075, USA; ^3^University of California Los Angeles, Los Angeles, CA 90095-1569, USA; ^4^RAND Corp., 1776 Main Street, Santa Monica, CA 90401-3208, USA; ^5^Southern California University of Health Sciences, Whittier, CA 90604, USA; ^6^Samueli Institute, 1737 King Street, Alexandria, VA 22314, USA; ^7^VA Long Beach Healthcare System, Long Beach, CA 90803, USA; ^8^Center for Health Policy Research, University of California, Irvine, CA 92697, USA

## Abstract

A systematic review was conducted to assess the level of evidence for integrative health care research. We searched PubMed, Allied and Complementary Medicine (AMED), BIOSIS Previews, EMBASE, the entire Cochrane Library, MANTIS, Social SciSearch, SciSearch Cited Ref Sci, PsychInfo, CINAHL, and NCCAM grantee publications listings, from database inception to May 2009, as well as searches of the “gray literature.” Available studies published in English language were included. Three independent reviewers rated each article and assessed the methodological quality of studies using the Scottish Intercollegiate Guidelines Network (SIGN 50). Our search yielded 11,891 total citations but 6 clinical studies, including 4 randomized, met our inclusion criteria. There are no available systematic reviews/meta-analyses published that met our inclusion criteria. The methodological quality of the included studies was assessed independently using quality checklists of the SIGN 50. Only a small number of RCTs and CCTs with a limited number of patients and lack of adequate control groups assessing integrative health care research are available. These studies provide limited evidence of effective integrative health care on some modalities. However, integrative health care regimen appears to be generally safe.

## 1. Introduction

In the United States, “institutional integrative health care (aka integrative medicine) is being developed in a highly individualistic manner (page 3)” [1]. Much of this literature refers to integrating CAM into institutional health systems and involves bringing CAM providers into conventional health centers [2]. However, there is another approach to integrative health care emerging within the CAM community itself, the insurance industry and with what is termed by one writer as the “CAM entrepreneurs” [2, 3]. 

There are various definitions and a diversity of terminology of integrative health care [4]. Maizes et al. define the term “integrative medicine” as “medicine that reemphasizes the relationship between patient and physician, and integrates the best of complementary and alternative medicine with the best of conventional medicine” [5]. Boon et al.'s [4] study on the working definition for integrative health care defines “integrative health care” as the combination of an interdisciplinary, nonhierarchical blending of both CAM and conventional medicine that employs a collaborative team approach guided by consensus building, mutual respect, and a shared vision of health through a partnership of patient and practitioners to treat the whole person by synergistically combining therapies and services in a manner that exceeds the collective effect of the individual practice [4]. Interestingly, at the prestigious IOM conference on integrative health care no formal definition emerged [6]. 

Therefore, the definition of integrative health care can range from simply incorporating CAM into conventional medicine to the notion that integrative health care constitutes a new form of medical practice. Stumpf, Shapiro, and Hardy refer to the malleable definitions of integrative health care as “one first step towards understanding the phenomenon.” However, a definition is more likely to emerge from key issues that are shaping the future of integrative medicine. That is, bilateral integrative medicine more resembles assimilation of CAM by biomedicine than true acculturation [7]. 

 It seems that regardless of the definitions, health care practitioners and policy makers have increasingly recognized that patients are using integrative health care to improve their wellness and treat illness [8]. Thus, for this paper, a guiding principle to the definition of *integrative health care research* is the study of the incorporation of CAM with biomedicine as a collaborative and integral part of the health care system. That is, the integration of conventional (allopathic) medicine and CAM, involving shared management of the patient, shared patient care, shared practice guidelines, and shared common values and goals to treat the well-being of the whole person.

There is preliminary theoretical and empirical data to support that integrative health care is not equivalent to CAM [9, 10]. However, the diversity of definitions, the ranges in levels of integration, and the degree of integration between CAM and biomedicine poses challenges to the practice, research, education, and policy/guideline decision and development in this evolving field. 

We posit that incorporation of CAM into conventional medicine without a joint management does not constitute integrative health care. In fact, the use of CAM as an adjunctive treatment to conventional care without a unifying paradigm or joint management may lead to worse outcomes because the selected CAM modality may adversely interact with conventional modality, and vice versa. In essence, integrative health care combines the best products and practices of CAM and conventional health care to optimize the body's natural healing processes [5, 11].

 The purpose of this paper is to review and summarize literature on Randomized controlled trial (RCT) and Controlled clinical trial (CCT), and systematic reviews on integrative health care as defined above and to make recommendations for future research on this topic.

## 2. Methods

We conducted a systematic literature review using a web-based, secure, systematic review management program called TrialStat SRS 4.0 (^©^Copyright 2003–2009 Mobius Analytics Inc, Ottawa, Ontario). TrialStat automates article progression and management, eliminates data transcription and reduces data collation work at the end of a review. This approach differs from conventional systematic literature review because it allows researchers to deliver results faster with improved accuracy and quality, providing a complete audit trail of all changes made in the systematic review. We searched the following databases from 1965 to Sept 2007: Pubmed, Allied and Complementary Medicine (AMED), BIOSIS Previews, EMBASE, the entire Cochrane Library, MANTIS, Social SciSearch, SciSearch Cited Ref Sci, PsychInfo, CINAHL, and NCCAM grantee publications listings. For the initial search, gray literature was searched using ProQuest Dissertations and Theses, and Google Scholar From our search strategy the keywords are identified in Table 3. 

 An updated secondary search was conducted in May 2009 during manuscript development to insure that all articles meeting the inclusion/exclusion criteria using the original search terms were included. In addition, hand searches and reference tracking were also performed, and the citation list was assessed for comprehensiveness by content experts. 

### 2.1. Inclusion and Exclusion Criteria

Our initial search terms were Integrat* and Medicine; Integrat* and Health* (for healthcare); multidisciplinary care; complementary or alternative and conventional medicine or health care; and delivery of health care and integrat*. 

Three investigators (RK, IC, and AH) independently screened titles and abstracts for relevance based on the inclusion criteria for this paper. Any disagreements about including a study were resolved through discussion and consensus. To avoid any misinterpretation, we excluded articles in languages other than English.

**Figure 1:**
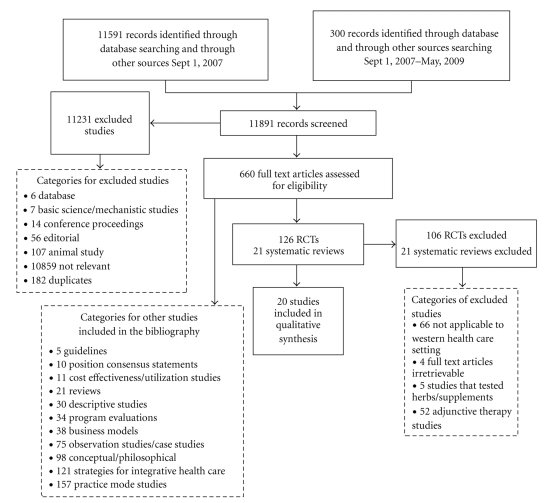
Systematic review flow chart.

This paper reports only on the RCT's, CCTs, and meta-analysis that passed our second level review screening. However, we have collected articles on practice models, models of integration, program evaluation, observational studies, cost-effectiveness, healthcare utilization, and so forth, which will be reported at a future date. The initial search retrieved all the above articles which were coded into their respective categories of study design. These were included in the bibliography but excluded from formal evaluation in this paper (see Figure 1).

Articles were included in this paper if they met the following criteria: (1) involved human subjects; (2) clinical study designs, including clinical controlled trials (including RCTs) and systematic reviews or meta-analysis involved with integrative health care research; and (3) integrative health care treatment as defined by the authors of this paper (including at least one modality from CAM and one modality from conventional medicine and they are combined using an integrative healthcare paradigm). We limited studies to those published in the English language. We also excluded studies with one of the following criteria: (1) did not present original data or an analysis of original data (i.e., commentaries, editorials, or expert opinion pieces); (2) were published in other media or in incomplete formats (i.e., abstracts, conference proceedings, posters, or web postings); (3) exclusively CAM without integration with conventional medicine; (4) use of CAM as adjunctive therapy to conventional medicine without incorporating an integrative health care paradigm in the design of the study; (5) studies that tested herbs and supplements; and (6) not applicable to Western health care setting. Also, articles on integrated care pathways, integrated review, integrated managed health system (i.e., electronic patient records, integrated delivery systems/networks or health care delivery), clinical integration, integrated case management, integrated analysis, exclusively CAM without conventional medicine integration, exclusively conventional medicine without CAM integration, scholarship of integration, integration into a curriculum for education, integrating theory of conventional medicine, integrated approach to concepts or integrating a single conventional medicine therapy to another, not applicable to western health care setting, and legalities or regulation of integration were excluded as “not relevant”.

This paper did not involve human or nonhuman experimentation and was exempt from Institutional Review Board (IRB) approval.

### 2.2. Methodological Assessment Process and Quality Rating

The methodological quality of the included studies was assessed independently by the reviewers (RK, IC, and AH). Each article was evaluated by type of study design and quality. 

RCTs, CCTs and meta-analysis were evaluated for study quality and study design bias using the Scottish Intercollegiate Guidelines Network (SIGN) checklists [12, 13] (see Table 1).

Also, there were independent assessments of the quality of the studies selected, and data extraction comprised descriptive information of the study population, the types of interventions, and the relevant outcome measurements and differences in ratings were resolved by reconciliation, discussion, and consensus. The outcome measures in each article were extracted and a determination was made regarding the direction of evidence for each study as strong, mixed, weak, or inconclusive (see Table 4). Controlled clinical trials (CCTs) (i.e., nonrandomized, pilot, single group, and other small studies) were qualitatively discussed in this paper regarding outcomes measures and study dropouts but not formally scored for bias such as randomization and blinding (see Table 4).

Total effect size was not calculated due to the heterogeneity of the trials identified during the review phase. All assessments were based on information provided in the published manuscripts that met the inclusion criteria.

### 2.3. Adverse Events Methodology and Reporting

Safety evaluation and adverse events reporting in all the RCTs were conducted using a 100 point safety assessment scale (SAS-CT) [14]. This scale has six major domains and the sub-domains have different weights according to their degree of importance and rate of occurrence. Safety evaluation and adverse events reporting in all the clinical studies was assessed by two investigators (RK and IC) and checked for consistency (see Table 2 for scoring information).

If all information required is presented in the text, tables, or figures, full points are given. If anything had to be extrapolated or added or half information is missing, half points are given. However, whether or not a conclusion can be drawn depends on the amount of information available [15] (see Table 4 for SAS-CT scores).

## 3. Study Selection

Our initial search yielded 11,591 citations from database inception to Sept 2007. An additional secondary search was conducted to capture studies published from September 2007–May 2009. This secondary search yielded 300 citations (see Figure 1).

## 4. Results on Quality Assessment and Trial Homogeneity

Heterogeneity across studies (i.e., outcomes, population, and the small number of studies per therapy) precluded formal meta-analysis. Therefore, it was only possible to conduct a descriptive analysis of the studies' design (study validity and bias) and outcomes reported.

 Of the 20 studies included in the qualitative synthesis, a total of 6 articles met the inclusion criteria of this paper. Of these 6 trials, there were 4 RCTs for which data were extracted in a standardized manner, and three independent investigators assessed the methodologically quality of RCTs using the (SIGN) checklist (see Table 1). Table 4 describes the main study characteristics and summarizes the findings of the 4 RCTs and 2 CCTs. The studies reviewed in this paper could not be combined for a total effect size because of the 4 RCTs, three (3) were conducted by the same research group on the same population and heterogeneity across studies. Using the SIGN 50, three of the 4 RCTs (75%) were rated as high quality “++”. One or (25%) of the RCTs was rated as neutral “+”, and no RCT was rated as low quality “−”. Individual SIGN item scores are available on Table 5.

## 5. Descriptive Results: Effects of Interventions

### 5.1. Clinical Trials on Multidimensional Integrative Health Care Interventions (*n* = 6)

Six articles [16–21] that were clinical trials of multidimensional integrative health care interventions were obtained. These 6 studies included four RCTs on integrative health care therapies to improve cardiovascular risk and/or disease symptoms [16–19], and two CCTs evaluating integrative health care therapies to improve quality of life/life satisfaction in women with breast cancer [20, 21]. Three of these RCTs were multiple reports published from the same research team on similar topics and populations—the efficacy of Noetic therapies before percutaneous intervention for unstable coronary syndromes [16–18].

The Monitoring and Actualization of Noetic therapy (MANTRA) clinical trials [16–18] examined the feasibility and efficacy of applying 4 Noetic therapies—stress relaxation, imagery, touch therapy, and prayer—to patients in the setting of acute coronary interventions compared to usual care. The results of two MANTRA trials, by Krucoff et al. [16, 17] were not statistically significant for any treatment outcomes comparisons. However, there was a reduction (25%–30%) in absolute incidence of major adverse cardiovascular events (i.e., mortality, myocardial infarction, congestive heart failure, and urgent or repeat revascularization) or adverse clinical events in patients treated with any Noetic therapies compared to usual care (control). Also, in the pilot study of the 4 individual Noetic therapies, off-site prayer was associated with the lowest absolute mortality in-hospital and at 6 months [16]. The parallel randomization to 4 different Noetic therapies across 5 study arms limited the assessment of possible synergy between therapies. 

 When the same research team examined the beneficial effects of Noetic therapies on mood assessed by Visual Analogue Scale (VAS) before percutaneous coronary intervention for unstable coronary syndromes they found that of the eight VAS scales on mood only one, [the VAS for worry], yielded significant effects (*P* < .05) in the stress management, imagery, and touch therapy groups compared to with usual therapy [18].

 The final RCT on an integrative health care to improve cardiovascular risk was based on multidimensional intervention principles [19]. Using a relationship-centered, mind-body approach (including mindfulness meditation, relaxation training, stress management, motivational techniques, and health education and coaching) in supporting behavior change, the study reported significant improvements in the 10-year cardiovascular risk as measured by the Framingham risk score (FRS) compared to usual care [19].

### 5.2. Two CCTs on the Choice of Care for Breast Cancer

Two nonrandomized studies, by Carlsson et al. [20, 21], compared quality-of-life of women with breast cancer who were treated with anthroposophic therapy (ABCW) after surgery for breast cancer, an individually composed therapies consisting of natural products, Iscador, diets, art therapy, eurhythmic therapy, therapeutic massage, hydrotherapy, compared to usual care. 

Carlsson et al. [20] conducted their first study to investigate the acceptability and feasibility of an anthroposophic therapy (ABCW) after surgery for breast cancer cared to usual care. They measured the differences in perceived quality-of-life/life satisfaction and coping strategies between the women receiving ABCW on entering the CCT compared to usual care alone. There were three primary outcome instruments used in this study: (1) the European organization for research and treatment of cancer quality-of-life questionnaire core 30 (EORTC QLQ-C30), (2) the life satisfaction questionnaire (LSQ), and (3) the differences in coping measured by the mental adjustment to cancer scale. The results found that women who choose ABCW reported lower quality-of-life and a greater psychosocial distress compared to the women receiving usual care on all measures on entering the study (*P* < .05). The dropout rate was about 13% for the ABCW group and 7% for the usual care group. The authors examined the possibility that perhaps the women in the ABCW group, who choose their individualistic care, might have been more independent personalities in choosing in their decision of care compared to the usual care group. A follow-up study reported that the women in ABCW group showed more passive and anxious coping on admission, but this decreased over time [22]. This could be one explanation for the ABCW higher dropout rate. 

A second study from the same team (2004) [21], found that women with breast cancer who participated in a 6-month CCT and 1 year follow-up reported a significant (*P* < .05) increase quality-of-life/life satisfaction (EORTC QLQ-C30 and LSQ) after ABCW treatment compared to the usual care alone. A follow-up study on the same population of women found that after 5 years, there were improvements in overall quality of life and in emotional and social functioning compared to admission for the women who choose ABCW. For these women, improvements took place between admission and 1 year, but not further on [23].

Table 4 summarizes the methodological details and results of these clinical trials of multidimensional integrative health care interventions.

## 6. Result Summary

Our initial search yielded 11,591 citations of which six (4 RCTs and 2 CCTs) [16–21] were trials of integrative health care interventions. We found that cardiovascular health and women's health were the two most common topics in integrative health care research. The total average age of adult subjects for the trial articles reviewed in this paper was 51.16 years. The most recent article in this paper was published in 2006, the oldest article was published in 2001. There were no studies on integrative pediatric care. 

Four RCTs were scored on internal validity and methodological bias using the SIGN checklist. Three of the 4 RCTs (75%) were rated as high quality “++”. One or (25%) of the RCTs was rated as neutral “+”, and no RCT was rated as low quality “−”. Half (50%) of the total clinical trials included in this paper were unable to report effectiveness of their intervention (i.e., no outcome measures as positive) despite sufficient power to do so.

 Most of these studies reported limitations such as the lack of credible controls for a placebo effect, inadequate assessment of long-term treatment benefits, and insufficient sample size, it would be difficult to generalize these findings. Two studies failed to report information on adverse events as required to be presented in the manuscript text, tables, or figures, and therefore scored 0 on the SAS-CT (see Tables 4 and 5).

## 7. Discussion

Two major conclusions can be drawn from this paper. The first is that there is an increasing, and somewhat extensive, body of literature with key terms Integrat* and Medicine; Integrat* and Health* (for healthcare); multidisciplinary care; complementary or alternative and conventional medicine or health care; and delivery of health care and integrat*. This search found over 10,000 articles. However, a second conclusion is that there are *very* few clinical controlled trials testing the efficacy of integrative health care. The large number of articles that were not relevant to our paper included studies on combining conventional services and alternative modalities without the incorporation of a collaborative and integral partnership of an integrated health care system. That is, the integration of conventional (allopathic) medicine and CAM, involving shared management of the patient, shared patient care, shared practice guidelines, and shared common values and goals to treat the well-being of the whole person. We found that the lack of a taxonomy makes searching this field very difficult.

This might reflect that this research topic is an emerging field. Like any emerging field integrative health care faces the problem of how to define and operationalize itself. That is, transforming the concepts into empirical and measurableentities. According to Maizes and Caspi, Integrative Health Care “requires a shift from the “fixing" paradigm that has been central to biomedicine (page 148)”. More importantly, it “shifts the paradigm from sickness to health, keeps the patient in the central focus of care, and multiplies the number of strategies available to the patient. It is a new kind of medicine that shifts the experience for both patient and provider (page 148)” [24].

Some have described Integrative Health Care as a transformative process [9, 25]. Mulkins's and Verhoef's study to identify factors for those patients who seek Integrative Health Care found 4 dimensions of transformation: (1) having access to a range of appropriate therapies to support individual journeys, (2) care that focuses on one's overall well being, (3) control over disease management, and (4) developing healing relationships with care providers [25]. Bell et al. also define Integrative Health Care as a transformative system represented by a higher-order system of systems of care that emphasizes wellness and healing of the entire person (bio-psycho-socio-spiritual dimensions) as primary goals, drawing on best both conventional and CAM approaches in the context of a supportive and effective physician-patient relationship [9]. While other proposed a continuum for team-oriented health care practice starting from the nonintegrative to fully integrative approach [26].

Therefore, to date *no* consensus has emerged about what constitutes Integrative Health Care. While there have been attempts to create outcome measures for Integrative Health Care (i.e., one-item visual analogue Arizona Integrative Outcomes Scale (AIOS), which assesses self-rated global sense of spiritual, social, mental, emotional, and physical well-being over the past 24 hours and the past month) [27] and there has been discussion on the importance of patient's perspective on integrative health care outcomes (i.e., physical well-being, change in physiological indicators, improved emotional well-being, personal transformation, feeling connected, global state of well-being, and cure) [10] none about what the important outcome variables are that *must* be measured. A growing body of literature documents the patient-based outcome assessment (PBOA) instruments that have been used to determine outcomes in CAM research to help practitioners and researchers make informed evidence-based decisions [28, 29]. However, we were unable to indentify similar literature for Integrative Health Care. Thus, the claimed inherent holistic nature of Integrative Health Care in which the social, psychological, spiritual, physical, and behavioral components oriented towards support and the stimulation of healing and the achievement of wholeness (i.e., the whole system) present special challenges with respect to design of clinical studies and especially with respect to the calculation of a total effect size. For this paper, we only reviewed clinical studies that involved a blending of both CAM and conventional therapy that provides a seamless continuum of decision-making and patient-centered care and support. However, heterogeneity across studies made it impossible to assess a total effect size.

The lack of trials might reflect the fact that for the most part the studies are of already existing programs in which it is not possible to set up controlled trials. Researchers here are for the most part studying programs that are already up and running so that the best that can be done is observational studies [30]. The most common situation for program evaluation is for existing programs so that we are comparing (often retrospectively) outcomes before and after implementation of the program [31]. 

Lastly, the lack for trial might also reflect that controlled trials may be an inappropriate research model here and that what are required are methods for whole systems research. “Thus, unlike biomedical research that typically examines parts of health care and parts of the individual, one at a time, but not the complete system, integrative outcomes research advocates the study of the whole. The whole system includes the patient-provider relationship, multiple conventional and CAM treatments, and the philosophical context of care as the intervention. The systemic outcomes encompass the simultaneous, interactive changes within the whole person (page 133)” [9].

Integrative Health care clearly involves, even allowing for differences in definition, the bringing together of differing complex systems of care [32]. By their very nature, they are not systems that lend themselves very readily to the type of research design required for controlled trials.

In spite of the nascent state of integrative health care research on clinical trials, clinicians may be able to use this literature review as a guideline for incorporating specific types of integrative health practices that are strongly evidence-based. There is still insufficient evidence to draw definite conclusions about the efficacy of integrative health care treatments; however, they appear to be generally safe. Available data suggest that some integrative health care therapies may warrant further study.

## 8. Limitations/Challenges Encountered in Reviewing the Field of Integrative Health Care

It was our goal to identify only published studies having to do with integrative health care. During the initial examination of key term and primary studies in this paper it was discovered that there was no clear terminology or definition for integrative health care. This lack of clarity made it difficult to generate search terms for the paper. For example, IM can turn up under integrated versus integrative medicine, integrated health systems versus holistic health systems. It might also appear under such things as traditional Chinese medicine and western medicine. To make sure we captured all these, the search terms by necessity had to be very broad. The downside of this strategy was that the search generated 11,891 total citations. Much additional work was required, therefore, to insure that the studies were truly focused on integrative health care. This has resulted in a level of review that is not usual when reviewing citations for inclusion. Further limitations included: (1) only studies in the English language were reviewed (2) both peer reviewed and nonpeer reviewed studies were reviewed. Nonpeer reviewed journals are generally considered of being lower quality.

 The two major limitations however are that we focused on clinical studies and only those that can be termed integrative health care as defined here. As noted earlier, this excluded most of the field. However, it is important to show that in the case of the strongest designs (i.e., RCTs and CCTs) and were it really is integrative, the field at the moment is very small.

## 9. Conclusions

There is still insufficient evidence from trials to strongly support the higher efficacy of integrative medicine regimen compared with usual care. However, integrative health care regimen appears to be generally safe. Additional high quality RCTs and CCTs are therefore needed to build a stronger evidence-based body of knowledge. This is the same recommendation that has been made for CAM [33].

## Funding Sources and Conflicts of Interest

No conflict of interest exists for any of the authors of this study. This paper is supported by the US Army Medical Research and Materiel Command under Award no. W81XWH-07-2-0076 through the Samueli Institute. The views, opinions, and/or findings contained in this paper are those of the author(s) and should not be construed as an official Department of the Army position, policy, or decision unless so designated by other documentation.

## Figures and Tables

**Table 1:** SIGN checklist [1].

Section 1: Internal validity*	

Item	Description

1.1	Study addresses appropriate, clearly focused question.
1.2	Treatment group assignment is randomized.
1.3	Adequate concealment method is used.
1.4	Subjects and investigators are kept “blind” about treatment allocation.
1.5	Treatment and control groups are similar at the start of the trial.
1.6	Only difference between groups is the treatment under investigation.
1.7	Outcomes are measured in a standard, valid, and reliable way.
1.8	What percentage of subjects in each treatment arm dropped out before the study was completed (i.e., record %)?
1.9	All subjects are analyzed in the groups to which they were randomly allocated (intention to treat analysis).
1.10	If the study is multisite, results are comparable for all sites.

Section 2: Overall assessment**	

How well was the study done to minimize bias? How valid is the study? Code ++, +, or −	

*Each item in Section 1 is to be evaluated using these criteria:	
(i)	Well covered
(ii)	Adequately addressed
(iii)	Poorly addressed
(iv)	Not addressed *(i.e., not mentioned, or indicates that this aspect was ignored) *
(v)	Not reported *(i.e., mentioned, but insufficient detail to allow assessment) *
(vi)	Not applicable

**The overall assessment uses the following ratings.	
++	*All or most* of the criteria have been fulfilled. Where they have not been fulfilled the conclusions of the study or review are thought *very unlikely* to alter.
+	*Some* of the criteria have been fulfilled. Those criteria that have not been fulfilled or not adequately described are thought *unlikely* to alter the conclusions.
−	*Few or no* criteria fulfilled. The conclusions of the study are thought *likely or very likely* to alter.

**Table 2:** SAS-CT Adverse Events Scoring.

The safety assessment score for controlled clinical trials (SAS-CT) consists of six major domains. Each domain and subgroup has different weightings according to its degree of important and rate of occurrence.
The maximum subscores of:
(i) adverse events definitely not related to the intervention (AEs-NR) and adverse drug reactions (ADRs) are 27 points,
(ii) serious adverse events definitely not related to the intervention (SAEs-NR) and serious adverse drug reactions (SADRs) are 13.5 points,
(iii) drop-outs due to AE/SAEs-NR and ADRs/ SADRs have a maximum subscore of 9.5 points each.

Total SAS-CT score per control clinical trial equals 100 points.

Quality is based on the degree of the importance of the subdomains.

The SAS-CT is separated into three quality levels:
(i) poor (0 < SAS-CT < 28)
(ii) medium (28 < SAS-CT < 68)
(iii) high quality safety reporting (68 < SAS-CT < 100)

**Table 3:** Official keywords as entered into the databases were as follows:

(i) (integrated or integrative) and medicine
(ii) (integrated or integrative) and health*
(iii) multidisciplinary care and alternative medicine
(iv) multidisciplinary care and complementary medicine
(v) (complementary or alternative) and conventional and (medicine or healthcare)
(vi) delivery of healthcare and (integrated or integrative)
(vii) (integrated or integrative) and patient evaluation
(viii) (integration and medicine) and (complementary or alternative)
(ix) (complementary or alternative) and medicine and (mainstream or biomedicine or orthodox).

**Table 4:** Descriptive of clinical trials of choice of care (multidimensional) integrative healthcare interventions (*n* = 7).

Reference	Design	Sample	Trial duration	Intervention	Control	Primary measures	Evidence direction on primary^#^	SIGN score*	Adverse events score
Krucoff et al. 2001 [16]	RCT	150	6 months	MANTRA	UC	DUREL, SSTA, Risk Stratification, and post-PCI ischemia	Weak	++	100
		Gender: 1 F							
		Mean Age: 63							

Krucoff et al. 2005 [17]	RCT	748	6 months	MANTRA: MIT or MIT plus prayer	UC or Prayer	DUREL, SSTA, and mood assessed by VAS before PCI for unstable coronary syndromes	Weak	++	100
		Gender: 214 F							
		Mean Age: 65							

Seskevich et al. 2004 [18]	RCT	150	15 months	MANTRA	UC	Mood assessed by VAS before PCI for unstable coronary syndromes	Mixed	++	0
		Gender: 1 F							
		Mean Age: 64							

Edelman et al. 2006 [19]	RCT	154	10 months	PHP	UC	10-year risk of CHD (FRS)	Strong	+	0
		Gender: 81 F							
		Mean Age: 53							

Carlsson et al. 2001 [20]	CCT	120	1 year	ABCW	CBCW	Life satisfaction (EORTC QLQ-C30, LSQ & MAC)	Weak	NA	100
		Gender: F							
		Mean Age: 49							

Carlsson et al. 2004 [21]	CCT	60	6 months plus follow-up	ABCW	CBCW	Life satisfaction (EORTC QLQ-C30 & LSQ)	Strong	NA	100
		Gender: F							
		Mean Age: 49							

Abbreviations: ABCW: Women with breast cancer, anthroposophic therapy; CBCW: Women with breast cancer, conventional therapy; CHC: Coronary heart disease; DUREL: Duke University religion index; EORTC QLQ-C30: European organization for research and treatment of cancer, quality of life questionnaire core 30; FRS: Framingham risk score; LSQ: Life satisfaction questionnaire; MAC: Mental adjustment to cancer scale; MANTRA: Monitoring & actualization of noetic training; MIT: Music, imagery, and touch; PCI: percutaneous coronary intervention; PHP: Personalized health planning; SMT: Spinal manipulative therapy; SSTA: Spielberger state-trait anxiety inventory; U: Unknown; UC: Usual care; and VAS: Visual analog scale.

^#^Evidence direction on primary measures. Weak: no outcome measures positive despite sufficient statistical power to do so, Mixed: at least one primary outcome measure positive at the level of *P* < .05, Strong ≥ 50% of measures positive at the level of *P* < .05, and Inconclusive: study failed to demonstrate a change but lacked the statistical power to demonstrate.

*SIGN checklist for RCTs and controlled clinical trials

++: Strong. All or most of the criteria have been fulfilled.

+: Article is neither exceptionally strong nor exceptionally weak.

−: Weak. Few or no criteria fulfilled.

**Table 5:** Items on internal validity*.

Reference	1.1	1.2	1.3	1.4	1.5	1.6	1.7	1.8	1.9	1.10
	Study addresses appropriate, clearly focused question.	Treatment group assignment is randomized	Adequate concealment method is used.	Subjects and investigators are kept “blind” about treatment allocation.	Treatment and control groups are similar at the start of the trial.	Only difference between groups is the treatment under investigation	Outcomes are measured in a standard, valid and reliable way.	What % of subjects in each treatment arm dropped out before the study was completed	All subjects are analyzed in the groups to which they were randomly allocated (intention to treat analysis).	If the study is multi-site, results are comparable for all sites.

Krucoff et al. 2001 [16]	Well Covered	Well Covered	Well Covered	Adequately Addressed	Well Covered	Well Covered	Well Covered	(1) MANTRA = 2%	Well Covered	Not Applicable

Krucoff et al. 2005 [17]	Well Covered	Well Covered	Well Covered	Adequately Addressed	Well Covered	Well Covered	Well Covered	(1) MIT + Prayer = 2%	Well Covered	Poorly Addressed
								(2) Prayer = 4%		
								(3) MIT = 6%		
								(4) UC = 5%		

Seskevich et al. [18]	Well Covered	Well Covered	Well Covered	Adequately Addressed	Well Covered	Well Covered	Well Covered	(1) MANTRA = 2%	Well Covered	Poorly Addressed

Edelman et al. [19]	Well Covered	Well Covered	Poorly Addressed	Poorly Addressed	Adequately Addressed	Adequately Addressed	Well Covered	(1) PHP = 27%	Well Covered	Not Applicable
								(2) UC = 14%		

Abbreviations: CMA: Conventional medical analgesia; EA: Electro-acupuncture technique; PCB: Paracervical block; MIT: Music, imagery, and touch; PHP: Personalized health planning; SMT: Spinal manipulative therapy; TMS: transcranial magnetic stimulation; and UC: Usual care.

*Each item in Section 1 is to be evaluated using these criteria:

(i) well covered,

(ii) adequately addressed,

(iii) poorly addressed,

(iv) not addressed *(i.e., not mentioned, or indicates that this aspect was ignored)*,

(v) not reported *(i.e., mentioned, but insufficient detail to allow assessment)*,

(vi) not applicable.
